# Patient centredness in integrated care: results of a qualitative study based on a systems theoretical framework

**DOI:** 10.5334/ijic.1361

**Published:** 2014-11-06

**Authors:** Daniel Lüdecke

**Affiliations:** Department of Medical Sociology, University Medical Center Hamburg-Eppendorf, Hamburg, Germany

**Keywords:** patient centredness, integrated care, DRG, systems theory

## Abstract

**Introduction:**

Health care providers seek to improve patient-centred care. Due to fragmentation of services, this can only be achieved by establishing integrated care partnerships. The challenge is both to control costs while enhancing the quality of care and to coordinate this process in a setting with many organisations involved. The problem is to establish control mechanisms, which ensure sufficiently consideration of patient centredness.

**Theory and methods:**

Seventeen qualitative interviews have been conducted in hospitals of metropolitan areas in northern Germany. The documentary method, embedded into a systems theoretical framework, was used to describe and analyse the data and to provide an insight into the specific perception of organisational behaviour in integrated care.

**Results:**

The findings suggest that integrated care partnerships rely on networks based on professional autonomy in the context of reliability. The relationships of network partners are heavily based on informality. This correlates with a systems theoretical conception of organisations, which are assumed autonomous in their decision-making.

**Conclusion and discussion:**

Networks based on formal contracts may restrict professional autonomy and competition. Contractual bindings that suppress the competitive environment have negative consequences for patient-centred care. Drawbacks remain due to missing self-regulation of the network. To conclude, less regimentation of integrated care partnerships is recommended.

## Introduction and problem statement

Patient-centred care has become an important aspect in health care delivery over the past years. It is seen as a core aspect of improving health care quality [1, 2]. The Institute of Medicine defines patient centredness as ‘health care that establishes a partnership among practitioners, patients, and their families (…) to ensure that decisions respect patients’ needs and preferences, and that patients have the education and support they need to make decisions and participate in their own care’ [3]. Nowadays, health care providers increasingly seek to improve patient-centred care in order to achieve better medical outcomes [4]. However, this goal cannot be achieved by single organisations solely. The integration of services and collaboration of health professionals and organisations is needed for improving health care quality and safety [5]. A primary reason why organisations in the health and social care sector should cooperate and implement integrated care concepts is the high fragmentation of support services and a strict separation of supply and care sectors. Health care providers are specialised in their tasks and offers so that patient-centred care and a comprehensive supply of care for people in need can only be established by collaboration and building integrated care partnerships [6, 7].

Organisations in the health care sector also have to manage costs for medical treatments. The challenge is to control costs, on the one hand, while enhancing the quality of health and medical care, on the other hand [8, 9]. Especially hospitals are affected by economic pressure due to a new remuneration system called Diagnosis-Related Groups (DRGs), which was invented in the USA and is implemented in many (European) countries [10, 11]. Since 2004, the costs for medical treatments in German acute hospitals are calculated according to this system. The former financing system covered the costs of how long a patient stayed in hospital, thus giving fewer incentives to discharge a patient as soon as possible. Now medical supply has to be achieved with limited financial resources due to the economic requirements from the DRG system. Hospitals have to stay within an average residence time for patients and leave further supply to other health care providers. Increasing costs and decreasing hospital-staying time require network-like co-operations. Thus, the DRG system fosters integrated care partnerships [12–14].

The challenge is to arrange and coordinate patient-centred care in a setting where many organisations are involved. Decisions on medical treatments, therapy options, in- or outpatient treatment, etc., are not made by a single central authority. Additionally, economic aspects may have an impact on these decisions.

Hence, the present paper addresses two questions: (1) How do integrated care networks effectively carry out control on collaborating health professionals and ensure patient-centred care under conditions of financial limitations due to the DRG system; (2) Which problems do collaborative networks solve with regard to patient centredness in health care delivery?

## Theoretical context

From a classical economic point of view [15], organisations and their structural characteristics can be located on a continuum between hierarchy and market. In the past, there was a ‘professional dominance’ (hierarchy) [16] in medical care where doctors decided on the completion of patients’ medical treatments and their hospital discharge. Handling the balance between medical and economical requirements was in the doctors’ authority [17–19]. This has changed dramatically with the introduction of the DRGs not only in Germany but also in many European countries [11], which led to a shift on the continuum from hierarchy towards market. The DRG system (market) ‘controls’ the discharge and transition planning in integrated care partnerships.

At first glance, it seems that nowadays market rules replaced hierarchy as predominating control mechanism in integrated care. However, having a closer look at organisational behaviour in integrated care partnerships, the hierarchy-market-continuum no longer seems to be suitable to describe the collaboration process. Organisations behave in a way that can be explained neither by referring to authority (hierarchy) nor by the logic of markets [20]. Rather, organisations are located in cooperation structures that rely much more on reciprocity, mutual interests and appraisal. Organisational behaviour cannot be sufficiently explained by either formal structures of hierarchy or profit-orientation. Powell suggests calling this control mechanism networks [20]. In this understanding, organisations appear as autonomous actors that cannot be controlled from outside [21]. This view is emphasised by systems theory research. Organisations, in general – and this applies to organisations within the health care and nursing sector as well, especially to hospitals, – can be seen as complex social systems, which have their own logic and dynamics [22, 23]. Systemic perspectives are helpful to get a better understanding of how collaborative networks ‘work’. The systems theoretical approach allows a comparison of networks and functional equivalent controlling mechanisms like hierarchical or market structures in order to underline advantages and disadvantages of different controlling mechanisms.

Unlike the Complex Adaptive Systems Theory (CAS) that considers systems as ‘open’ [7], in this paper, the systems theory by Niklas Luhmann [24] is used as theoretical framework to analyse the cooperation of organisations, supplemented by aspects of Powell's [16] and White's [25] network theory approaches. Luhmann adopted the concepts of self-organisation and self-reference of systems [26, 27] which means that systems are operationally closed. This perspective was emphasised later when Luhmann also integrated the autopoiesis concept, invented by the biologists Maturana and Varela [28]. Autopoiesis explains how a cell can reproduce itself without direct input from its environment, since the cell itself is separated from the environment via its membranes. Luhmann applied the concept of operational closure on social systems, which reproduce themselves by the basic operation of communication.

The concept of operational closure explains why control attempts (for instance, implementing the DRG system to control quality and costs in integrated care) do not automatically change an organisation's behaviour according to the primary goals of health politics. This problem becomes even more virulent in the context of integrated care: Decisions and routines made in one organisation are not automatically applied to or accepted by other organisations. There is no central instance or authority for controlling (steering) the integrated care and cooperation process. Different organisational structures have to be taken into account. In collaborative networks, each party has certain expectations of what the other network partners can or should do, and what can be expected from oneself.

In this sense, from a systems theoretical view, networks can be considered as attempts to develop, form and control identities and enforce own interests [25]. Organisational behaviour, as an attempt to form and control its identity, is influenced and ‘limited’ by other organisations’ attempts of controlling network-processes and vice versa. The network relations are self-conditioned by mutual observations. From this point of view, organisations no longer appear as purpose-rational institutions that are defined by their members, which pursue own interests. Rather, organisations are ‘purpose-seeking systems’ [29, 30] that evolve independent from its members’ actions and motives. The principle of self-organisation is a central point in this definition of ‘networks’.

## Methods

The research interest of this paper was to investigate the control mechanism of collaborative networks in the context of integrated care partnerships and how this mechanism affects the challenge of high quality in health care delivery under limited financial resources. To pursue this question, a qualitative approach was chosen that facilitates access to the implicit knowledge and the social practise of health professionals in collaborative networks [31]. The data that will be analysed for this paper stem from qualitative, semi-structured interviews. An interview guide covering the topics of integrated care, patient centredness, barriers and facilitators of collaboration as well as economic aspects was developed and pre-tested. Seventeen persons in different acute hospitals of metropolitan areas (Hamburg, Bremen and Osnabrück) have been interviewed. Twelve of the seventeen hospitals were privately operated. Five were NPO. Due to the logistic effort of personal face-to-face interviews, the sample region was restricted to northern Germany. The sample size was chosen according the criteria of saturation. While about fifteen interviews are generally recommended for this kind of qualitative studies [32], after seventeen conducted interviews, no further insights into the issue under investigation were revealed and hence the stopping criterion for sample saturation was reached. The questioned persons are either case managers or social workers and work in the field of hospital discharge, i.e. their professional context is embedded into integrated care partnerships and interface management. None of these persons belonged to the nursing staff. The duration of interviews varied between 45 and 60 minutes. They were digitally recorded and transcribed with respect for keeping mentioned persons anonymous. Parts of the interviews were evaluated and interpreted collaboratively in data analyses workshops with colleagues.

According to Vogd [33] and Bohnsack et al. [34], the documentary method as a qualitative reconstructive research approach seems suitable to analyse organisational behaviour in collaborate networks. Following this method, the researcher tries to reconstruct the modus operandi of (organisational) practice. The question is, how social formations emerge, which means, the focus lies on the process of ‘sense–making’ in organisations [35, 36]. Three analytic steps are involved: the formulating interpretation, the reflecting interpretation and the constant comparative analysis.

In the first step of formulating interpretation, the researcher chooses specific passages of a text for further in-depth analysis. These passages are identified according to thematic relevance, where a possible interview-guideline can be used as rough framework to retrieve the formal structure of an interview. During this analytic step, the researcher tries to reformulate these passages in order to gain an overview of relevant topics for the following interpretation. The next two steps, reflecting interpretation and constant comparative analysis, are performed simultaneously in practise. In the reflecting interpretation, the aim is to identify different ways of how a specific problem is handled. By contrasting passages in different interviews or even different passages within the same interview that deal with certain problems, latent pattern of organisational structures are identified. This analysis step requires a constant comparative analysis. The analysed passages serve as comparing perspectives that identify the ‘contrast of similarities’ [37] and allow for methodological control, as the implicit knowledge of the actors becomes more and more explicit. ‘Consequently the case comparison is to ensure the inter-subjectification of the results’ and reduces ‘the risk that the interpreters are trapped in their own expectations of normality and thus approach the data with cultural norms that do not do them justice’ [38]. In case of sequential analytical approaches like that suggested by the documentary method, computer-aided data analysis (i.e. software like MaxQDA) does not provide much benefits over a ‘pen-and–paper’ procedure [39]. Thus, the data analysis was carried out by directly working with the text documents.

## Results

The following data analysis focuses on the structures of integrated care partnerships. It emphasises how the involved health care providers put patient centredness and quality of care into practice.

### Patient-centred perspectives

Collaborating with health care professionals in the context of hospital discharge planning requires mutual reliability and support. Partners develop experiences in good working co-operations that allow estimating which quality of service provision may be expected. Based on this experience, they try to find the appropriate service provider for the follow-up care of patients:

I really want to see the patient in good hands. And I think, since then we have developed a sure instinct which provider fits to a patient. For instance, we have partners that are very good in supplying junkies. They really get along very well with them. However, this would not be the best provider for an old, anxious grandma. And this works really well, the concession to saying: we look after the administrative stuff and we expect from you that you'll take our patient. And this is clear for both sides. (I5)

The co-operation is based on respect and on trust. Hence, mutual expectations are established which ensure an informal way of working together. With regard to the network partners, the interviewed person talks about expectations (‘we expect …’) and not about obligations. The expectations are ‘clear for both sides’, which means, mutual reliability has been developed. Patient centred care benefits from this kind of collaboration because it is known that the patient is ‘in good hands’.

Integrated care partnerships are based on a mutual give-and-take basis where one party is dependent on resources controlled by another. Playing the game of integrated care means the health providers agree not to pursue own interests at the expense of others. In this context, patient centredness can be claimed.

I would say, it should be more like a give-and-take basis, so you can say ‘I benefit from knowing other organisations, where I have the feeling that my patient is very well accommodated there. He receives high quality care, his individuality is respected.’ And in critical situations, we all know of our good collaboration, and all provide patient centred care. (I8)

Of course, health care professionals may differ in their understanding and scope of patient-centred care. The quality of patient-centred care must not be inevitably similar among co-operation partners. However, network structures may foster the quality due to mutual control, expectations and demands.

Well, we think of a work group with all our co-operation partners in which ethical problems are discussed. How we can deal with these problems and how to improve our collaboration according to this topic. For instance, nutrition of nursing and care home residents. There's a strong tendency to apply a PEG (percutaneous endoscopic gastrostomy). Now, what defines a good co-operation in this case? To say, the hospital inserts a PEG and the patient can go back into the nursing home and is fed? To think of alternatives how to deal with such situation in order to consider the patient's needs and wishes? Who is having the benefit? The patient, or the health provider? (I4)

In such case, one health professional can demand patient centredness to a certain extent from other partners. A common agreement on minimum requirements regarding patient-centred care has to be found in order to start cooperation. Negotiations of tasks in integrated care partnerships are guided by characteristics of patient-centred care.

It is interesting to see that patient-centred care is not only limited to the context of concrete treatments (e.g. taking patients’ wishes and preferences into account), but can also play a role in the context of how network structures are understood. Patient centredness can also be achieved indirectly as a ‘by-product’ of good collaboration.

If we look at the patient, and this is of course a little bit in my focus as well, the benefit from good working co-operation is that the patients feel much more comfortable, safe and in good hands when they recognize that all professionals that are involved in treatment or therapy know from each other. And that all professionals are on the same level of information and do not ask the same diagnostic questions again. For patients it is a huge advantage if we perform a good collaboration. (I2)

It is important to point out that especially the mutual expectations with regard to patient-centred care foster the structures of cooperation. In this case, there is no need for contractual bindings that define the criteria of health care quality. Good collaborations can, of course, also involve contractual bindings, which also may be beneficial in terms of patient centredness.

In our contract-based co-operation we collaborate with three or four partners. The advantage is that we can perform our work more structured and precisely. We do not need to search for an appropriate provider. (I7)

However, contract-based cooperation is not purely positive. According to patient centredness, a contract-based integrated care concept may limit choices, where better alternatives have to be omitted due to contractual obligations.

The other side of the coin: contractual bindings affect the patients’ choice of alternatives. From our perspective, there are good reasons to have a variety of uncommitted network partners with no contract-based relationship. Where we get feedback, and thereby can evaluate their work. So we know how good they are, thus we can pick up a partner from a larger pool of providers. (I7)

From the interviewees’ perspectives, integrated care partnerships that are embedded in loosely coupled network structures without hieratic regimentation offer a permanent evaluation of quality criteria. In case someone drops below a minimum quality standard in health care provision, other network partners may no longer ‘have the feeling that my patient is very well accommodated there’.

Of course, we're interested in in arranging the co-operation to find a common basis, where we have the feeling that the collaboration works well. If other providers mainly look at economic aspects, we just say that we can't recommend them to patients and that we are not willing to collaborate. (I3)

### Economic perspectives

Especially hospitals perceive a strong pressure in the context of patient treatment due to the DRG remuneration system. The hospital staying length becomes the primary factor, which affects the discharge planning and the following collaboration. Patients have to be discharged in time if hospitals do not want to risk financial penalties. An interviewed person describes the DRGs’ impact on his work:

It is great for the workflows. According to the workflows, we do not plan them on a gut level nor just for medical reasons. We also have to take into account the DRG remuneration when it comes to co-operation and integrated care, because the DRG system dictates us an average hospital length of stay for each patient. To comply with these limits is our duty because it's required from the health care insurance companies. We have to respect this. Sometimes this situation is not what we want. And sometimes it's against the patients’ wishes. And sometimes, quite rarely, it's also against our company's opinion. (I16)

The DRG system becomes a structural element that ‘embeds’ the integrated care partnership into a timeframe wherein the coordination and collaborative tasks have to be accomplished. Although hospitals are obliged to cope with this remuneration system, which makes the discharge planning challenging, it is still considered as positive (‘It is great for the workflows’) because it can be used as a pressurising medium to control and optimise the workflows. At a first glance, the scope of actions are limited due to the tight time frame set by the DRGs, but it actually offers new opportunities of professional autonomy and control.

Anyway, reasonably shaping or structuring the workflows according to all concerns: patient wishes and orientation, clinical pathways, a constant high-level medical treatment of patients and the pinpoint of hospital discharge, all these are supported by a successful co-operation. Apart from that, a harmonic rapport has positives effects on the working atmosphere that leads to less workload. I can simply call and say ‘hey pal, I have someone for you for admission, you already know him …’ That's much easier than any bureaucratic procedures. And working like this simply makes more fun. (I16)

On the one hand, the DRG system exerts economic pressure on hospitals. This circumstance calls for well-planned and precise workflows that influence the collaboration of the team by establishing specific structures, which define the scope of action. On the other hand, uncomplicated and unconstrained ways of cooperation are preferred. At a first glance, this argumentation seems to be contradictorily, but it makes sense in the context of decision-making and professional autonomy. The DRG system serves as ‘controlling frame’ that ensures (voluntary) liability of network partners with no need for contractual bindings, leaving enough autonomy and freedom for decision-making to all collaborating health care professionals. These kinds of loosely connected networks without bureaucratic barriers allow for fluent workflows.

### Collaborative perspectives

Most notably, almost all questioned health professionals underline the importance of informality. In their habitual self-conception, case managers think of cooperation as flexible and self-organising network structures.

We have working groups with different topics, with a certain circle of people who regularly meet again. And this is really sustainable co-operation. Because due to this co-operation we see and know each other, we can discuss problems informally with no need for bureaucratic procedures. This really fosters stable co-operations. (I12)

Informal negotiations are preferred rather than contractual obligations. This ensures that professional autonomy of all providers in integrated care partnerships is kept. Mutual reliability eases the cooperation. In this understanding, networks rely on expectations and reliability. They are rarely regulated. By fathoming out mutual expectations, these structures become stable. Regimentations, however, are rejected and considered as disadvantageous.

It's something completely different if a good, informal co-operation changes into tight contractual bindings with a limited amount of companies that seal themselves off from the others. Well, you may see advantages like an information highway for patients’ data in such cases. But the disadvantages in contractually bound co-operation preponderate quite clearly. (…) If a good co-operation leads to exclusivity, it has very negative impacts on the market and causes considerable perturbations among the competitors. It also narrows the scope of action we have. Co-operation is great and structuring workflows are great as well. But exclusive co-operations – only you and no one else – that's nothing, I don't like that. So, in this point I like to keep this boundary of my work untouched. (I9)

Here we can see some interesting aspects. ‘Good’ and ‘bad’ cooperations are described, while only the ‘good’ one is explicitly named as such. ‘Bad’ cooperations, however, seem to be the result of the shift from informal to exclusive, regimented cooperation, which is considered as negative. Thus, contractual bindings in integrated care are not favoured. Finally, the reasons for this understanding are lack of competition and limiting the scopes of action health professionals have. This clearly emphasises the importance of professional autonomy in integrated care partnerships for the involved actors. Lose couplings in networks ensure flexibility and keep spheres of influence for the involved network partners.

In such an understanding, cooperation networks do not need contractual regulations; they are ‘self-regulating’ or ‘self-organising’. The mechanism for regulation works on a ‘give-and-take basis’. This indicates a balanced power relationship between the partners.

On the one hand, we can realize short-dated discharge of patients. And on the other hand, I think, well, they get new customers from us and thus the other providers have financial benefits from these patients. I think, this can be considered as mutual give-and-take. (I11)

As said before, a typical characteristic for networks is the network partners’ mutual dependency of each other's resources. This seems to be a core aspect of controlling integrated care partnerships. It allows for the freedom of choice to which cooperation relationship the health professionals commit. For instance, if a potential co-operation partner does not fulfil certain standards of patient-centred care, a new partner might be chosen. Network structures based on contracts, however, do not offer this flexibility and professional autonomy.

Well, we are not depending on the follow-up service providers in order to get new patients, not at all. Rather, our co-operation partners or the many nursing homes, they want our patients! And every now and then they give us a call to say, “hi there, we're still there and we offer this and we have that services.” – Yes, like, “we haven't received any patients from you for so long, how does it look like right now?” – Or they call to tell us how many free beds they have, and so. – And we say, “nice to hear from you, we'll inform our colleagues about this.” (I11 & I12)

This shows that networks appear as an own mechanism to control integrated care partnerships and ensures the self-organisation of such systems.

## Discussion

The core question of this paper was how professional health care providers deal with the problem of high quality in health care delivery under conditions of economic limitations (costs) and in which way collaborative networks (integrated care) can contribute to solve this problem.

From the empirical findings, we can derive that economic limitations especially affect the hospitals according to their discharge planning. There is no option getting more payments if a patient stays longer in hospitals. Increasing costs and decreasing hospital-staying time require network-like cooperations. These findings are emphasised by other studies that show how this remuneration system fosters the necessity of cooperation and coordination in health care delivery [12, 13]. However, looking at the data, it does not seem that the pure logic of market is a suitable instrument to control the integrated care process. The same seems true for hierarchic structures with clearly defined rules that determine the tasks and workflow in cooperation networks. All questioned health professionals have similar ideas of characteristics of integrated care partnerships. They point out the informal character of the relationship of network partners. They all are against consortiums and contractual relations. Networks, in their understanding, consist of ‘loosely coupled’ elements. This correlates with a systems theoretical conception of organisations, which are assumed autonomous in their decision-making due to operational closure [23]. Self-regulation (or self-organisation) of organisations (autonomous decision-making and operational closure) needs loosely coupled networks. First, this ensures that no conjoint binding rules, which can be considered as control attempts from the outside, work against a system's autopoiesis (survival). Second, lose couplings ensure high flexibility in networks [40].

These findings are in line with other studies that relied on systems theoretical frameworks. ‘Change cannot be forced and attempts to control the system or prescribe innovation are often counterproductive due to the potential to destabilize the system’ [7]. Rather, integrated care partnerships should rely on structures that ensure self-organisation. This kind of control mechanism is what Powell defines as ‘networks’ [20]. In this spirit, following the idea of Åkerstrøm Andersen [41], networks can be considered as ‘reflexive contract’. Reflexive awareness, mutual control and professional autonomy are the basis for sustainable network structures and keep these structures stable. Profit-seeking consortiums do not have the necessary reflexive awareness and adaptability. They may have a negative impact on patient centredness because inappropriate conditions and mal-supply cannot be eliminated by self-regulation. In this sense, collaborative networks can solve the problem of missing self-organisation. Therefore, integrated care partnerships should be based on autonomy (from the systems or organisations perspective) in the context of reliability (from the networks or environmental perspective).

Patient-centred care can be achieved in such kinds of integrated care partnerships. The possibility of alternatives according to the choice of network partners seems to be a tool for regulating mal-conditions. If two health providers cannot find a common basis for their relationship because they may completely differ in their understanding of patient-centred care, other network partners will be chosen. To remain attractive as network partner and benefit from health care delivery (in terms of making money), health providers have to behave cooperatively in a way that each party in the network has to stick to the ‘reflexive contract’. The interviewed health professionals called this a ‘give-and-take basis’ for their relationship. Networks appear as reflection strategy according to the stability and durability of integrated care networks – also taking into account the point of staying in a network. From a network theoretical perspective, the health professionals’ behaviour can be described as interplay of ‘identity and control’ [25]. They enforce their claims towards other network partners, negotiate tasks in the context of hospital discharge planning and fathom advantages and disadvantages (identity). They reflect reactions from other network partners and adjust their future behaviour in order to stay in the collaborative network (control).

## Limitations

This study uses a relatively small sample of case managers and social workers in the context of integrated care. It is difficult to say how far these findings are transferable to integrated care partnerships in general. Interviewees were mostly involved in informal collaborations. For deeper analyses of advantages and disadvantages of contract-based cooperation, other cases should be included in the sample. Moreover, the sample was taken from metropolitan regions of Germany, where many health care providers exist and compete. In rural areas, there are often less alternatives for choosing cooperation partners, so network structures may differ in such regions. Finally, only the perspective from the hospital staff was included in the data analysis. The recommendation for further research is to enhance the ‘network perspective’ by contrasting different views of all involved professional providers.

Nevertheless, even having a limited sample size – which is, however, typical for qualitative study designs [32] – the findings are based on a reconstructive research method. This type of method allows conclusions on specific social patterns even in case of smaller sample sizes, given that the steps of data analysis follow the rules of the applied methodological approach [34].

## Conclusion

In conclusion, the recommendation from the perspective of systems and network theory as well as the empirical data is to be careful when planning integrated care based on formal contracts and hierarchical structures. Limiting the professional autonomy, limiting the competition and implementing inflexible co-operation structures may have negative impacts on patient-centred care and health care quality. Contractual bindings in networks that suppress the competitive environment may have negative consequences for patient-centred care. This finding may not apply in general to all formalised integrated care partnerships. However, there are empirical examples that show how hierarchical structures led to malpractices in patients’ medical treatment and its related financial charging. In the so-called ‘Helios affair’ in Berlin [42, 43], the competition of health providers was limited by regimented network structures that included just a few actors. This affected the quality of patient provision because missing reflexive awareness and self-regulation of the network could not eliminate this drawback.

Collaborative networks based on informal relationships solve this problem of missing ‘feedback system’. Evaluation criteria in terms of good patient centredness evolve from the reciprocity and self-organisation in such networks. Hence, reflexive awareness in networks becomes a quality indicator that promotes health care quality and patient centredness. The same applies to competition, which also improves the quality of care [44]. This is also because health care delivery embedded in formalised hierarchical structures provokes a behaviour, which mostly has a negative impact on the patients’ autonomy of decisions [45].

Integrated care partnerships may benefit from relying on networks considered as reflection strategy based on professional autonomy in the context of reliability. Contract-based collaboration concepts therefore should define quality standards and include methods to ensure feedback and evaluate the work of network partners.

In this sense, ‘the parties to a network agree to forego the right to pursue own interests at the expense of others’ [20]. This may lead to improved health care quality and better patient-centred care.

## References

[1] Charmel PA, Frampton SB (2008). Building the business case for patient-centered care. Healthcare Financial Management.

[2] Weijden Tvd, Légaré F, Boivin A, Burgers JS, Veenendaal Hv, Stiggelbout AM (2010). How to integrate individual patient values and preferences in clinical practice guidelines? A research protocol. Implementation Science.

[3] Hurtado MP, Swift EK, Corrigan JM (2001). Envisioning the national health care quality report [Internet].

[4] Luxford K, Safran DG, Delbanco T (2011). Promoting patient-centered care: a qualitative study of facilitators and barriers in healthcare organizations with a reputation for improving the patient experience. International Journal for Quality in Health Care.

[5] Cunningham FC, Ranmuthugala G, Plumb J, Georgiou A, Westbrook JI, Braithwaite J (2012). Health professional networks as a vector for improving healthcare quality and safety: a systematic review. BMJ Quality and Safety.

[6] Toscan J, Mairs K, Hinton S, Stolee P (2012). Integrated transitional care: patient, informal caregiver and health care provider perspectives on care transitions for older persons with hip fracture. International Journal of Integrated Care.

[7] Tsasis P, Evans JM, Owen S (2012). Reframing the challenges to integrated care: a complex-adaptive systems perspective. International Journal of Integrated Care.

[8] Zismer DK, Werner MJ (2012). Managing the physics of the economics of integrated health care. Physician Executive.

[9] Zhao L-P, Yu G-P, Liu H, Ma X-M, Wang J, Kong G-L (2013). Control costs, enhance quality, and increase revenue in three top general public hospitals in Beijing, China. PLoS ONE.

[10] Wiley M (2011). From the origins of DRGs to their implementation in Europe. Diagnosis-related groups in Europe moving towards transparency, efficiency and quality in hospitals.

[11] Quentin W, Scheller-Kreinsen D, Blümel M, Geissler A, Busse R (2013). Hospital payment based on diagnosis-related groups differs in Europe and holds lessons for the United States. Health Affairs (Millwood).

[12] Stock S, Plamper E, Redaèli M, Gerber A, Lauterbach KW, Klauber J, Robra B-P, Schnellschmidt H (2005). Versorgungspolitische Ziele der Integrierten Versorgung. Wege aus der Krise der Versorgungssituation. Beiträge aus der Versorgungsforschung.

[13] Vogd W, Mozygemba K, Mümken S, Krause U, Zündel M, Rehm M, Höfling-Engels N (2009). Braucht die neue Medizin das Subjekt? Überlegungen zur Organisation der Krankenbehandlung im Zeitalter des New Public Management. Nutzerorientierung - Ein Fremdwort in der Gesundheitssicherung?.

[14] Ham C (2012). Competition and integration in health care reform. International Journal of Integrated Care.

[15] Williamson OE (1983). Markets and hierarchies: analysis and antitrust implications: a study in the economics of international organization.

[16] Freidson E (2007). Professional dominance: the social structure of medical care.

[17] McKinlay JB (1977). The business of good doctoring or doctoring as good business: reflections on Freidson's view of the medical game. International Journal of Health Services.

[18] Freidson E (1980). Doctoring together: a study of professional social control.

[19] Hafferty FW, Light DW (1995). Professional dynamics and the changing nature of medical work. Journal of Health and Social Behavior.

[20] Powell WW, Godwyn M, Gittell JH (2011). Neither market nor hierarchy. Network forms of organization. Sociology of organizations: structures and relationships.

[21] Bakken T, Hernes T (2003). Autopoietic organization theory: drawing on Niklas Luhmann's social systems perspective.

[22] Brunsson N (1985). The irrational organization: irrationality as a basis for organizational action and change.

[23] Luhmann N, Bakken T, Hernes T (2003). Organization. Autopoietic organization theory: drawing on Niklas Luhmann's social systems perspective.

[24] Luhmann N (1996). Social systems.

[25] White HC (2008). Identity and control. How social formations emerge.

[26] Foerster HV, Zopf GW (1962). Principles of self-organization: transactions.

[27] Foerster HV (1976). Objects: Tokens for (Eigen-)behaviors. ASC Cybernetics Forum.

[28] Maturana H, Varela F (1980). Autopoiesis and Cognition: The Realization of the Living.

[29] Luhmann N (2000). Organisation und entscheidung.

[30] March JG, Olsen JP (1979). Ambiguity and choice in organizations.

[31] Nohl A-M, Bohnsack R, Pfaff N, Weller W (2010). Narrative interview and documentary interpretation. Qualitative analysis and documentary method in international educational research.

[32] Mason M (2010). Sample size and saturation in PhD studies using qualitative interviews. Forum Qualitative Sozialforschung/Forum: Qualitative Social Research [Internet].

[33] Vogd W, John R, Henkel A, Rückert-John J (2010). Methodologie und Verfahrensweise der dokumentarischen Methode und ihre Kompatibilität zur Systemtheorie. Die Methodologien des Systems Wie kommt man zum Fall und wie dahinter?.

[34] Bohnsack R, Pfaff N, Weller W (2010). Qualitative analysis and documentary method in international educational research.

[35] Weick KE (1995). Sensemaking in organizations.

[36] Weick KE, Sutcliffe KM, Obstfeld D (2005). Organizing and the process of Sensemaking. Organization Science.

[37] Bohnsack R, Pfaff N, Weller W, Bohnsack R, Pfaff N, Weller W (2010). Reconstructive research and the documentary method in Brazilian and German educational science. An introduction. Qualitative analysis and documentary method in international educational research.

[38] Evers H (2009). The documentary method in intercultural research scenarios. Forum Qualitative Sozialforschung/Forum: Qualitative Social Research [Internet].

[39] Weiß A (2010). Erfahrung in Auswertung Dok.Meth. mithilfe von MAXqda? [Internet].

[40] Baecker D, Baecker D (2007). Zur Krankenbehandlung ins Krankenhaus. Wozu Systeme?.

[41] Åkerstrøm Andersen N (2008). Partnerships: machines of possibility.

[42] (2011). B.Z./dapd. Helios-Klinik:Betrug? Razzia bei Helios-Klinik. BZ-online [Internet].

[43] Bach I, Radke J (2011). Verdächtige Helios-Ärzte weiter im Dienst. Der Tagesspiegel Online [Internet].

[44] Fornaciari D (2010). Quality health care in the European Union thanks to competition law. International Journal of Environmental Research and Public Health.

[45] Dahm F-J (2008). Unzulässiger Kooperationsvertrag zwischen Krankenhaus und Arzt. Der Urologe A.

